# Research on the spatial patterns and evolution trends of the coupling coordination between digital finance and sustainable economic development in the Yellow River Basin, China

**DOI:** 10.1371/journal.pone.0296868

**Published:** 2024-01-08

**Authors:** Qiguang An, Yongkai Wang, Ruoyu Wang, Qinggang Meng, Yunpeng Ma

**Affiliations:** School of Statistics and Mathematics, Shandong University of Finance and Economics, Jinan, China; Qufu Normal University, CHINA

## Abstract

In the current global context, digital finance (DF) and sustainable economic development (SED) are important topics. The synergies between DF and SED have already been proven. However, the measurement and quantitative analysis of the coupling coordination degree (CCD) of DF and SED have not received sufficient attention to date. Based on data from 55 cities in the Yellow River Basin (YRB) from 2011 to 2021, this study constructs an evaluation index system of DF and SED and measures their level, respectively. The proposed CCD model is then used to measure the CCD between the two systems. In addition, kernel density estimation, Markov chain, *σ*-convergence, *β*-convergence, and the quadratic assignment procedure (QAP) method are used to study the spatial pattern, distribution dynamic evolution trend, convergence, and influencing factors of the regional differences in the CCD. The results show that: (1) From 2011 to 2021, the CCD level showed a stable upward trend and regional heterogeneity, and the time stage characteristics were more obvious. (2) The center position and change interval of the overall distribution curve of the kernel density estimation gradually shifted to the right. The Markov transfer probability matrix shows that the CCD is more stable among different levels, indicating a phenomenon of “club convergence”. (3) A convergence analysis shows that there are significant *σ*-convergence, absolute *β*-convergence, and conditional *β*-convergence. (4) The QAP regression shows that factors such as the regional differences in GDP per capita have a significant impact on the regional differences in the CCD. This study offers a comprehensive structure that can be used to examine the synergistic effects between DF and SED; the research findings can also provide perspectives for other areas.

## 1 Introduction

Climate change and the rise in global temperatures have created a serious environmental problem. Globally, the annual generation of municipal waste already amounts to 1.3 billion tons, a figure that is projected to reach 2.2 billion tons by 2025 [1]. This waste generation has profound implications for human health. In 2020, China proposed the “double-carbon” target, committing to peak carbon dioxide emissions by 2030 and to achieve carbon neutrality by 2060. The “double-carbon” target, however, poses certain challenges to China’s economic development, such as higher production costs and lower economic growth. In light of the “double-carbon” target, how to realize sustainable economic growth is already an important issue for the Chinese government and will continue to be so in the coming period.

As an engine of social development, finance plays a pivotal role in sustainable economic development (SED). In the era of big data, digital finance (DF) has also made significant strides. The concept of DF belongs to the same category as Internet finance, financial technology, etc. Essentially, DF is a new business model in which traditional financial institutions and Internet companies utilize technologies such as big data, blockchain, and other technologies to achieve financial product innovation [2]. In February 2023, the Chinese government issued the “Overall Layout of Digital China Construction Planning”. The document maintains that the comprehensive digital transformation of the financial industry will be realized, so that better financial services can be provided for the development of the real economy. The development of DF can effectively address the limitations of traditional finance and provide financial support for innovative activities, thus increasing investment in innovative research. At the same time, the development of DF is conducive to guiding the flow of social capital to the advanced manufacturing industry. This will help to optimize the allocation of resources and will consequently foster green and sustainable development in both society and the economy.

The Yellow River Basin (YRB), which is both rich in natural resources and densely populated, spans three major regions in eastern, central, and western China [3]. The YRB traverses nine provinces in China, stretching across a total length of 5,464 kilometers and an area of 795,000 square kilometers [4, 5]. Ensuring the sustainable development of the YRB effectively contributes to building a foundational power source for China’s overall sustainable development. In 2021, the Chinese government issued the Outline of the Plan for the Ecological Protection and High-Quality Development of the YRB. This programmatic document serves as a guiding framework for the YRB’s environmental protection, providing a crucial foundation and roadmap for SED in the region. Therefore, this paper selects the cities along the YRB as a sample, measures the coupling and coordination degree (CCD) of DF and SED, and analyzes the cities’ spatial and temporal evolution and convergence. This analysis aims to provide a better understanding of the level of coupling and coordination between DF and SED and also aims to identify the dynamic trends in promoting green, low-carbon, and sustainable societal development. By doing so, this study offers a theoretical basis for the policy recommendations aimed at enhancing the coupling and coordination between DF and SED.

Without question, DF and SED are dialectically unified, and there exists a coupling mechanism of mutual promotion and coordination. On the one hand, DF can promote SED. This is particularly evident in the inclusive impact of DF, given its wide coverage and extensive utilization. Nowadays, DF can expand the coverage of financial services, DF can also effectively guide the flow of financial resources to small and micro enterprises, the “three rural” sectors and other key areas, addressing their existing weaknesses. By doing so, DF enhances financial accessibility and promotes SED. Conversely, SED serves as the foundation for the development of DF. With the continuous development of the economy and society, both the Internet and China’s physical infrastructure have made great progress, these advances have laid the foundation for the birth of DF and at the same time, the dissemination of DF. The development and popularization of the economy and society are inextricably linked to the sustainable development of the economy and society. At present, the research on the relationship between DF and SED primarily concentrates on the following four aspects:

Firstly, there is the impact of DF on environmental improvement, especially on carbon emissions reduction. Some studies have found that DF has an environmental inclusion effect, which can help create the win-win situation of economic development and environmental protection [6]. Also, DF can significantly improve China’s energy and ecological performance, in which case green technology innovation becomes the transmission path for DF to influence energy and environmental performance [7]. In addition, with regard to the impact mechanism of DF on carbon emission efficiency, several studies have indicated the existence of a U-shaped relationship. During the initial stage of DF development, an inverse correlation exits between DF and carbon emission efficiency, and DF’s green effect has not yet appeared. However, with the continuous maturity of the development of DF, the green effect brought about by DF will continue to increase in intensity, and this will make up for the effect of pollution brought about by DF [8, 9]. Some scholars have also used spatial econometric modeling to conduct empirical research on the impact of DF on carbon dioxide emissions. The results show that, from a spatial perspective, DF can promote urban carbon emission reduction [10].

Secondly, there is the relationship between DF and enterprise sustainable development or high-quality development. Many previous studies have shown that DF can facilitate the sustainable development of companies. The main influence mechanisms include improving the competitive environment of banks, reducing the cost of financing, and fostering the innovation ability of enterprises. Most of the studies in this category have discussed samples of listed companies [11, 12]. Other studies have also focused on specific types of enterprises, such as the influence mechanism of DF, as well as the high-quality development of state-owned companies [13] and the impact of DF on the sustainable development of small and medium-sized companies [14]. The conclusions of the above studies are similar, in that they all indicate that the application of DF or digital technology has a certain role in fostering the sustainable development of companies.

Thirdly, there is the relationship between DF and the sustainable development of other industries, such as manufacturing and agriculture. Some researchers have shown that the booming development of DF in China effectively promotes the upgrading of the manufacturing industry. In addition, DF has a significant positive effect on the servitization of China’s manufacturing industry [15]. Regarding agriculture, some studies have shown that DF can directly promote the high-quality development of agriculture, while also indirectly contributing to the development of agriculture through factors such as the level of agricultural mechanization, thereby fostering the sustainable development of agriculture. At the same time, DF can also foster the sustainable development of rural areas through the channels of economic efficiency, urban-rural structure, and green ecological development [16, 17].

Finally, there is the research on the relationship between DF and the sustainable development of the regional economy. Most such studies have focused on aspects such as DF and regional economic innovation. For example, with the data from 31 of China’s provinces, Hui et al. (2023) [18] studied the effect of DF on the capability for innovation. Meanwhile, a small number of scholars have conducted research on the coupling and coordination relationship between DF and SED. Using a CCD model based on data from Chinese provinces, Ma et al. (2023) [19] analyzed the CCD and convergence characteristics of DF and the development of the advanced manufacturing industry. Using provincial data of China, Wu and Ang (2022) [20] innovatively explored the relationship between DF, green finance, and ecological governance.

**Fig 1:**
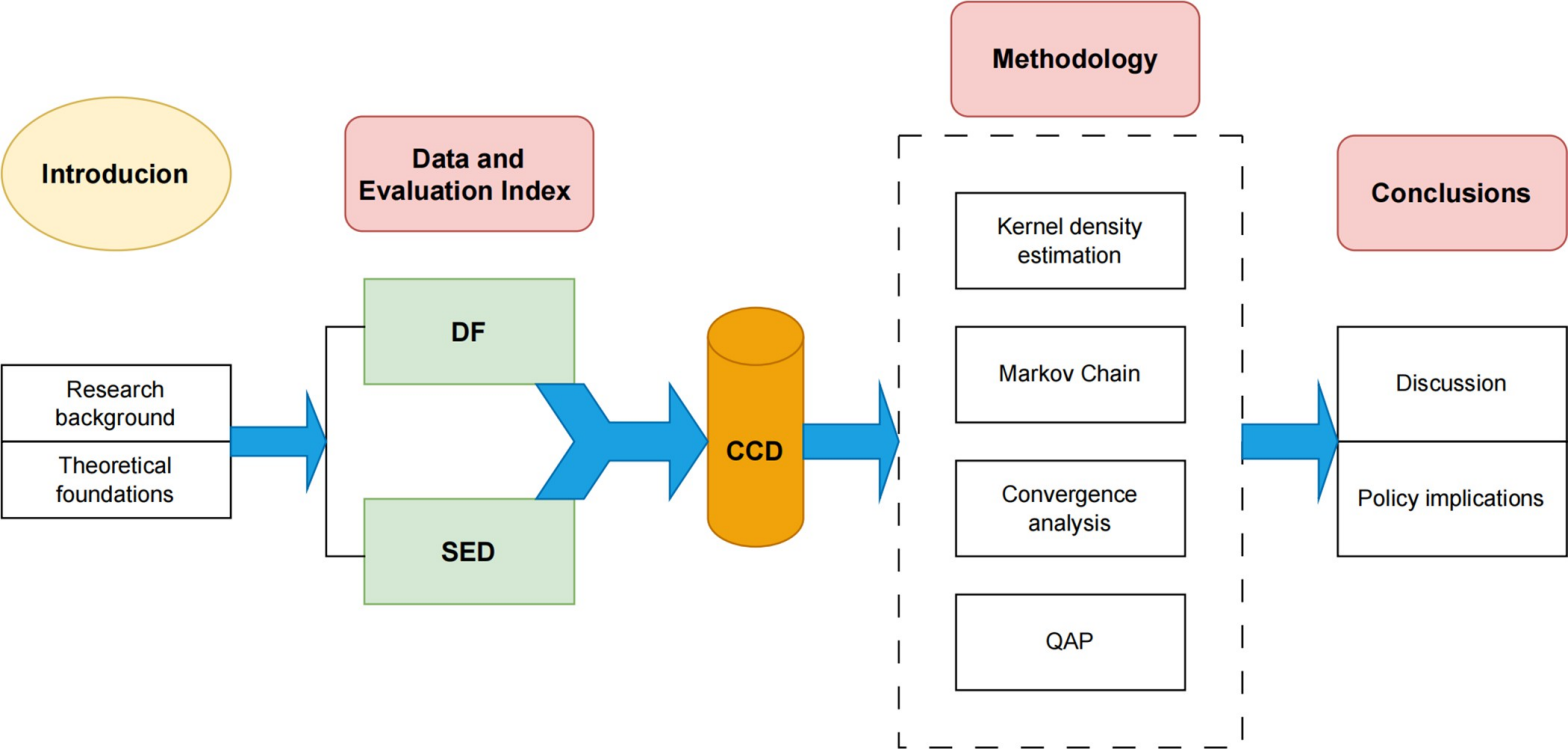
The framework of this paper.

After a systematic combing of existing literature, no literature has been found that has examined the coupled and coordinated relationship and convergence between DF and SED in the YRB. Given this gap in research, this paper takes the cities of the YRB as a sample to research the coupling and coordination characteristics and convergence of DF and SED. Firstly, the evaluation index system of DF and SED is constructed separately; the entropy method is also utilized to gauge the level of the two systems. Secondly, the proposed CCD model is applied to measure the CCD. In addition, kernel density estimation, Markov chain, *σ*-convergence and *β*-convergence methods are comprehensively applied to analyze the temporal and spatial differences in the distribution and dynamic evolution trends and convergence of the CCD. Finally, the quadratic assignment procedure (QAP) method is employed to empirically test the influencing factors of regional differences in CCD. The framework of this paper is shown in Fig 1.

## 2 The evaluation index system and methodology

### 2.1 Study area

**Fig 2:**
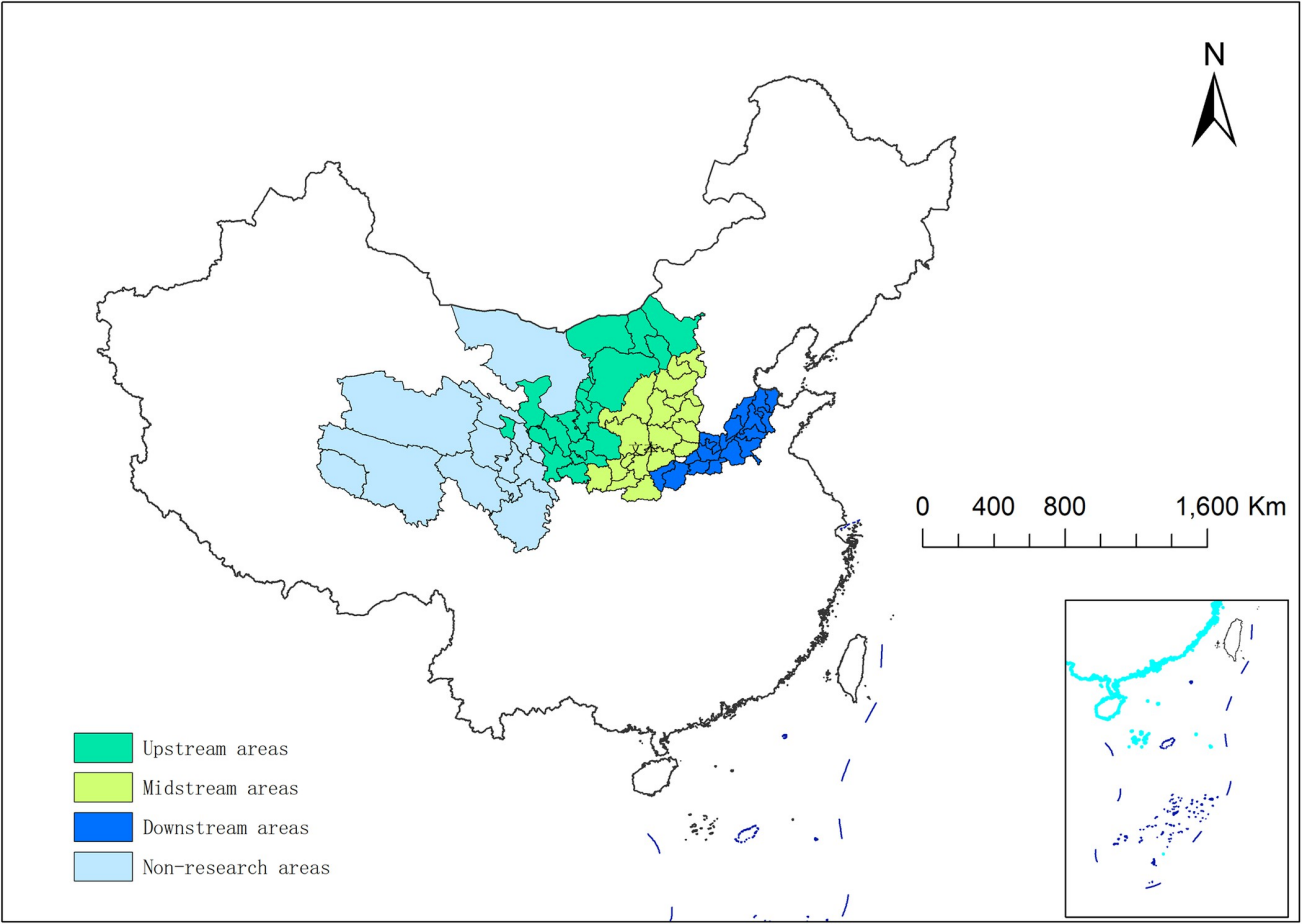
Specific distribution location of the YRB in China. The map is based on the standard map with review number GS (2019) 1822 downloaded from the Standard Map Service website of the Ministry of Natural Resources (http://bzdt.ch.mnr.gov.cn/), with no modifications to the base map.

Regarding the division and selection of specific cities in the YRB, this research is based on the division of specific cities in the Chinese government’s “Outline of the YRB Ecological Protection and High-Quality Development Plan” and the relevant documents formulated by each province, respectively. This study also refers to previous relevant studies [21]. In all 66 prefectural-level cities were selected, of which 11 cities were not examined, due to serious data deficiencies. Meanwhile, the sample cities were divided into three regions: upstream (18 cities), midstream (19 cities), and downstream (18 cities). Their specific locations in China are shown in Fig 2.

Since the digital inclusive finance index was first released in 2011, and because some data at city level for 2022 are unavailable, the sample interval of this paper is set as 2011–2021. The data for digital financial indicators come from the digital inclusive finance index. The data for the indicators of SED, such as invention patents, trademark registrations, and attractiveness of foreign investment, come from the Index of Regional Innovation and Entrepreneurship in China (IRIEC) published by the Center for Enterprise Research of Peking University. The data for the remaining indicators come from the China Urban Statistical Yearbook, the CSMAR regional economic database, and the CNRDS database. The missing data of some years and cities have been manually collected and organized from regional statistical bureaus, government work reports, etc. Missing data at the individual level have been imputed using interpolation techniques. See S1 Dataset.

### 2.2 Construction of evaluation index system

In terms of the assessment criteria for DF and SED, within the academic community, there is currently no universally agreed-upon standard. Various scholars have developed indicators based on their own specific research objectives, and this approach has led to a lack of consensus. Regarding the evaluation indicators of DF, two main approaches have been employed. The first use a single indicator, such as the scale of third-party payments or regional Internet penetration rate, as a proxy indicator for DF. This method may have the problem of poor representativeness and may not accurately reflect the current DF situation. The second, and currently the most used in the academic community, is the digital inclusive finance development index constructed by the Peking University Institute of Digital Finance [22]. The index is compiled based on the transaction account data of Ant Gold Service Group. The data not only contain a comprehensive index of digital finance in each city in China but also describes the level of DF in three dimensions, namely breadth of coverage, depth of use, and digitization level. While most existing studies have directly used the China digital inclusive finance development index, this paper innovatively chooses the three dimensions of breadth of coverage, depth of use, and digitization level. In addition, referring to the method of Wang et al. (2022) [23], the entropy value method is used to measure DF, specifically because the three dimensions of DF can more comprehensively and accurately measure the development of DF.

Numerous scholars and studies have explored the connotation and construction of an evaluation index system for SED. Relevant studies can be broadly classified into the following categories: First, one category is based on the concept of new development [24, 25]. Studies of this kind mainly construct evaluation index systems based on the five dimensions of innovation, coordination, green development, openness, and sharing. Building upon these five dimensions, Deng et al. (2023) [26] added the “economic” dimension, resulting in a total of six dimensions, to construct a comprehensive set of indicators for assessing high-quality economic development. The study used this system to evaluate the extent of high-quality economic development in the Jing-Jin-Ji city cluster. Li and Liu (2022) [27] introduced the dimension of “safety”. The study also constructed an evaluation index system for the high-quality development of China’s marine fisheries from the six dimensions of “openness, innovation, coordination, greenness, sharing and safety”. Second, from the standpoint of economic expansion, scholars have typically used indicators such as GDP [28], GDP per capita [29], total factor productivity [30], green total factor productivity [31, 32], labor productivity [33] and other indicators as proxy variables for SED and research on the factors that affect SED. The advantages of this type of research are that the calculation of indicators is relatively simple. However, such an approach also faces the problems of poor representativeness, one-sidedness, and limitations of the indicators, which cannot comprehensively describe the quality of SED. Third, other multidimensional characterization perspectives have been represented by green development. For example, Zhang et al. (2022) [34] used the undesirable-SE-SBM model to calculate green development efficiency. This study refers to the studies of Han et al. (2023) [35], Yin et al. (2023) [36], and Zhang et al. (2023) [37] to construct an evaluation index system of SED from the five dimensions of economic vitality, innovation power, green development, open development, and shared development. These dimensions contain 19 secondary indicators, such as the rationalization of the industrial structure, the advancement of industrial structure, and other indicators. The specific evaluation index system for DF and SED is shown in Table 1.

**Table 1:** Evaluation index system of DF and SED.

System layer	Target layer	Indicator layer	Unit	Direction	Weight
DF	Breadth of coverage	Breadth of coverage	Score	+	0.3252
	Depth of usage	Depth of usage	Score	+	0.4071
	Digitization level	Digitization level	Score	+	0.2677
SED	Economic vitality	Rationalization of industrial structure	Score	-	0.0069
		Advancement of industrial structure	%	+	0.0531
		Per capita regional GDP	Yuan	+	0.0516
		Risk control	%	-	0.0028
	Innovation power	IRIEC patent license number score	Score	+	0.0346
		IRIEC trademark registration number score	Score	+	0.0442
		Investment in science and technology	%	+	0.0471
		Human capital	%	+	0.1789
	Green development	Industrial smoke and dust emissions	Tons/10^4^ yuan	-	0.0056
		Industrial sulfur dioxide emissions	Tons/10^4^ yuan	-	0.0124
		Non-hazardous treatment rate of domestic waste	%	+	0.0168
	Open development	IRIEC city FDI attraction score	Score	+	0.0338
		Number of foreign-invested enterprises	Number	+	0.1759
		Urban attraction	%	+	0.0329
	Shared development	Rural to urban income ratio	%	+	0.0620
		Hospital beds per 10,000 population	Number of beds	+	0.0394
		Per capita parkland area	Square meters	+	0.1240
		Quantity of books in public libraries	Volume/person	+	0.0438
		Urbanization rate	%	+	0.0344

### 2.3 Research methods

#### 2.3.1 Entropy method

As a widely-used objective assignment method, the entropy method is more objective and scientific as a dynamic comprehensive evaluation method. The entropy method’s main concept is to take the information entropy of each index as the weight and arrive at comprehensive evaluation results through weighted averages. The outlined procedures are as follows:

In view of the fact that the unit of measurement of each indicator is not uniform, to mitigate the potential impact that may be caused by different quantitative outlines between the indicators, the initial step involves standardizing the raw data of the DF and SED.

$$Positive\;indicators:\;r_{ij}=\frac{x_{ij}‐\min(x_{ij})}{\max(x_{ij})‐\min(x_{ij})}$$ (1)

$$Negative\;indicators:\;r_{ij}=\frac{\max(x_{ij})-x_{ij}}{\max(x_{ij})‐\min(x_{ij})}$$ (2)

Where *x*_*ij*_ is the original data, and *r*_*ij*_ is the index value after dimensionless processing; *i* represents the evaluation system, and *j* represents the specific evaluation indicators (*j* = 1,2,⋯,*m*). Then, the entropy weight method is used to determine the weight of each indicator, and the formula for calculating the comprehensive development index of each system is as follows:

$$U_{i}=\sum_{j=1}^{n}w_{j}r_{ij}$$ (3)

Where *U*_*i*_ is the comprehensive development level of each system and *j* = 1,2,⋯,*n* denotes the count of indicators contained in each system.

#### 2.3.2 Dual-system coupling coordination model

In the field of physics, coupling denotes the phenomenon during which two or more systems jointly affect each other through interaction. A CCD model can be used to evaluate the degree of coordination between different systems and can truly reflect the degree of coupling between different systems and system effects [38]. In this paper, a coupling coordination model of DF (*U*_1_) and SED (*U*_2_) is constructed. The model is then used to examine the coordination effect and dynamic evolution characteristics of DF (*U*_1_) and SED (*U*_2_), and the specific measurement model is as follows:

$$C=\sqrt{\frac{U_{1}\times U_{2}}{{\left(\frac{U_{1}+U_{2}}{2}\right)}^{2}}}$$ (4)

$$D=\sqrt{C(\alpha U_{1}+\beta U_{2})}$$ (5)

In the model, *U*_1_ and *U*_2_ represent the level of DF and SED, respectively; *C* denotes the system’s harmonization level, ranging from 0 to 1, which indicates the extent of alignment in the interplay between DF and SED. Finally, *D* denotes the CCD of DF and SED, taking the value of [0,1]. A higher value corresponds to a greater degree of coupling between DF and SED, as well as a closer the relationship between the two systems to promote each other and coordinate their development.

**Fig 3:**
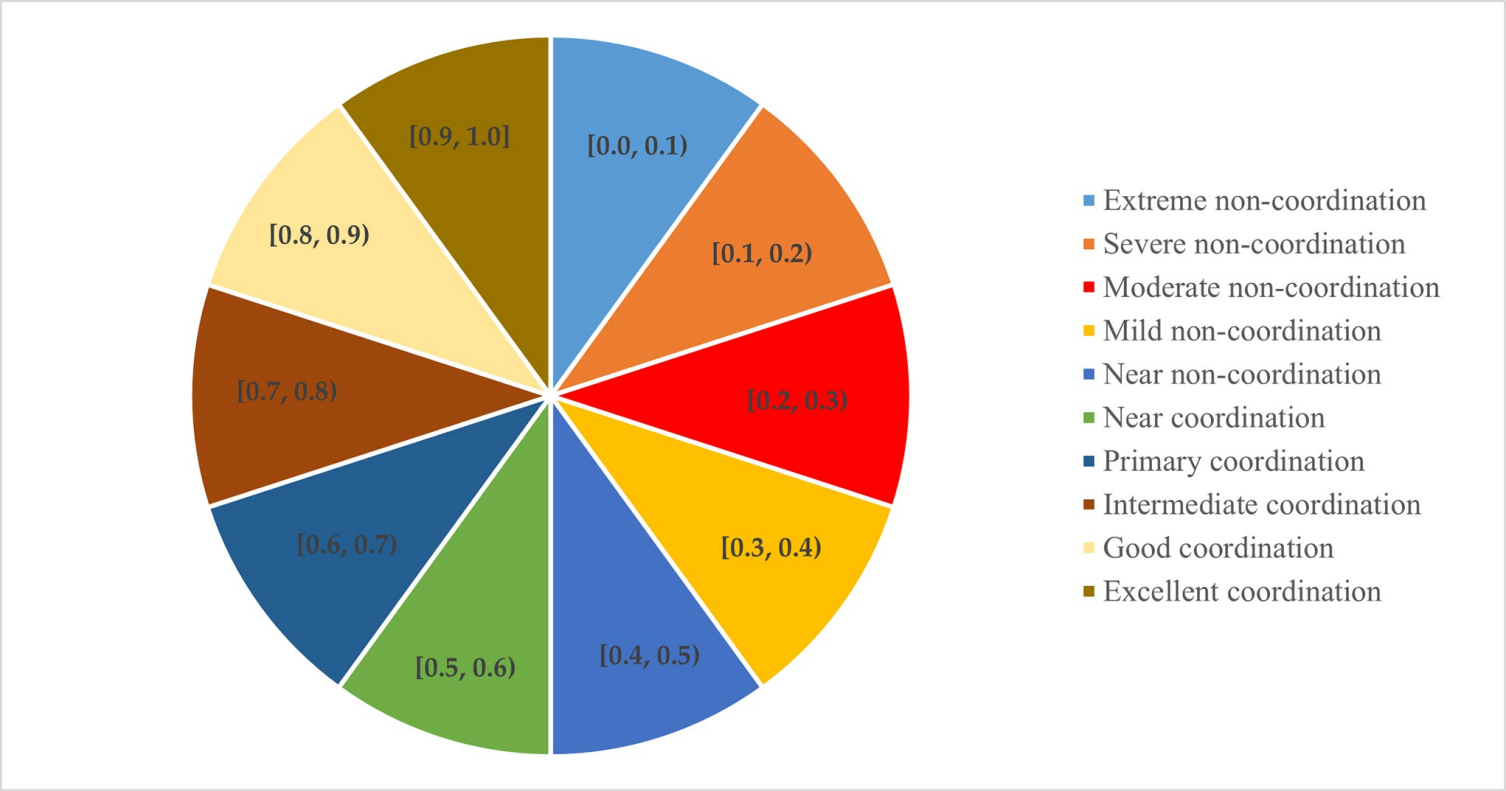
The classification of CCD.

To more intuitively reflect the CCD between DF and SED, this study draws on the research of Chen et al. (2023) [39] to classify CCD between the two systems into the 10 grades shown in Fig 3.

#### 2.3.3 Kernel density estimation

In this study, the kernel density method is employed to analyze the distributional dynamics of the CCD of DF and SED in the YRB. The kernel density function for the random variables can be expressed as:

$$f(x)=\frac{1}{nh}\sum_{i=1}^{n}K(\frac{\bar{x}-x_{i}}{h})$$ (6)

Where *K*(•) is the kernel function, *X*_1_,⋯*X*_*n*_ is the CCD of the cities in the sample, $\bar{x}$ is the mean value, *n* represents the total number of sample observations, and *h* is the window width. In this study, the high-precision Gaussian kernel function is chosen to estimate the dynamic distribution level of the CCD for DF and SED. The functional expression is:

$$K(x)=\frac{1}{\sqrt{2\pi }}e^{\left(-\frac{x^{2}}{2}\right)}$$ (7)

#### 2.3.4 Markov chains

To depict the distribution and evolutionary patterns across various temporal and state categories, Markov chains are employed, which discretize continuous values into distinct types. First, the traditional Markov chain is utilized to analyze the internal trend characteristics of the system’s CCD. In addition, the quartile method is employed to divide the CCD into the four grades of low, medium-low, medium-high, and high. This is done according to their sizes on average and to measure the transfer probability matrix. Second, based on the spatial correlation test, a spatial Markov transfer probability matrix (MTPM) is established. A spatial Markov chain can effectively compensate for the neglect of neighboring regions’ interactions in the traditional Markov chain method by combining it with the concept of “spatial lag”. Here, the weighted average attribute value of neighboring regions is calculated by adding a spatial weight matrix to judge the spatial lag condition of regional units [40].

**2.3.5 Convergence analysis.** *(1) σ-convergence*. To measure the convergence characteristics of the CCD of DF and SED in the YRB, the coefficient of variation is introduced to conduct a *σ*-convergence test. The trend of the coefficient of variation of the CCD of DF and SED in the three regions and the YRB as a whole is observed over time, and judgements are made regarding the intraregional differences have convergence. The calculation formula is as follows:

$$CV_{it}=\frac{\sigma_{it}}{{\bar{D}}_{it}}=\frac{\sqrt{\frac{1}{m_{i}}\sum_{m=1}^{m_{i}}{\left(D_{imt}-{\bar{D}}_{it}\right)}^{2}}}{{\bar{D}}_{it}}$$ (8)

Here, *σ*_*it*_ denotes the standard deviation of region *i* in year *t*, *D*_*imt*_ denotes the coupling coordination level of the city *m* and region *i* in year *t*, *m*_*i*_ represents the count of cities included in region *i*, and ${\bar{D}}_{it}$ represents the means of the CCD in region *i* and year *t*.

*(2) β-convergence*. This paper adopts *β*-convergence to examine the evolution trend of the CCD of DF and SED in the YRB. Derived from neoclassical growth theory, *β*-convergence theory is divided into absolute *β*-convergence and conditional *β*-convergence. A spatial correlation analysis reveals a significant positive correlation between the coupling and coordination level of DF and SED in the YRB. Traditional standard convergence theories do not consider the spatial dependence among regions. Hence, this paper utilizes a spatial adjacency weight matrix for spatial convergence analysis. In conducting a series of tests, such as LM, Hausman, and LR, this paper selects a two-way fixed-effects spatial Durbin model (SDM) to analyze both absolute and conditional *β*-convergence. The model is formulated as follows:

$$\ln(\frac{q_{i,t+1}}{q_{i,t}})=\alpha +\beta \ln(q_{i,t})+\rho W_{ij}\ln(\frac{q_{i,t+1}}{q_{i,t}})+\gamma W_{ij}\ln(q_{i,t})+\mu_{i}+\eta_{t}+\varepsilon_{it}$$ (9)

$$\begin{array}{l} \ln(\frac{q_{i,t+1}}{q_{i,t}})=\alpha +\beta \ln(q_{i,t})+\sum_{z=1}^{n}\lambda_{z}X_{it}+\rho W_{ij}\ln(\frac{q_{i,t+1}}{q_{i,t}})+\gamma_{1}W_{ij}\ln(q_{i,t})\\ \;+\sum_{z=1}^{n}\gamma_{2z}W_{ij}X_{it}+\mu_{i}+\eta_{t}+\varepsilon_{it} \end{array}$$ (10)

Where *q*_*it*_ denotes the coupling and coordination level of DF and SED in city *i* in period *t*, $\ln(\frac{q_{i,t+1}}{q_{i,t}})$ represents the growth rate of the CCD of city *i* in period *t*, and *β* is the convergence coefficient. If *β*<0, this means that a convergence trend exits in the level of coupling coordination of DF and SED, and vice versa. The convergence trend is divergent, and the rate of convergence is *υ* = −ln(1+*β*)/*T*; *T* is the length of the study period, the halfway convergence period *τ* = ln(2)/*υ*. *ρ* is the spatial lag coefficient, and *W*_*ij*_ is the spatial adjacency weight matrix. Then, *μ*_*i*_, *η*_*t*_, *ε*_*it*_ represent area-fixed, time-fixed effects, and random error terms, respectively. Finally, *X*_*it*_ represents the selected control variables. The analysis framework of the factors that influence the CCD of DF and SED is mainly constructed from five aspects: GDP per capita, urbanization rate, advancement of industrial structure, number of software employees, and market openness.

#### 2.3.6 Quadratic assignment procedure (QAP)

Regional differences in CCD can be regarded as a kind of “relationship” between regions; the variables in the form of relational data often have autocorrelation problems and serious multicollinearity. Therefore, traditional statistical test methods will no longer be applicable. This paper examines the influencing factors of regional differences in CCD from a relational data perspective. Specifically, a resampling-based QAP method is used to reveal the driving forces behind them. The relational data analysis paradigm is now introduced in terms of measurement model setting and QAP.

*(1) Modeling*. The measurement model for relational data adopted in this study is as follows:

$$Y=\beta_{0}+\beta_{1}X+\varepsilon$$ (11)

Where *Y* is the dependent variable, i.e., regional differences in CCD; *β*_0_, *β*_1_ is the parameter to be estimated, *X* is the explanatory variable (specifically, the main factors that may affect regional differences in CCD), and *ε* is the residual term. One can see that, in the form of the measurement model, the relational data model and attribute data measurement model are consistent. The difference is that the former variable data form is an n-order square matrix.

*(2) QAP*. In practice, QAP is mainly used to test the correlation between networks [41]. This method does not need to assume that the variables are independent of each other, and QAP can effectively avoid the autocorrelation and multicollinearity problems of data measurement models. The difference between relational data measurement models and general data measurement models is that the variables in the former model are in the form of ordinal matrices. Also, some scholars currently utilize the basic principles and advantages of the QAP relational data analysis paradigm to construct difference matrices to explain the formation of differences. Within the QAP framework, correlation analysis and regression analysis are employed [42]. Correlation analysis focuses on examining the interrelationships between two matrices, while regression analysis investigates the predictive relationship between multiple matrices and a single matrix.

## 3 Analysis of regional differences and the dynamic evolution of CCD

### 3.1 System CCD and regional differences

#### 3.1.1 Measurement and analysis of the level of DF and SED

**Fig 4:**
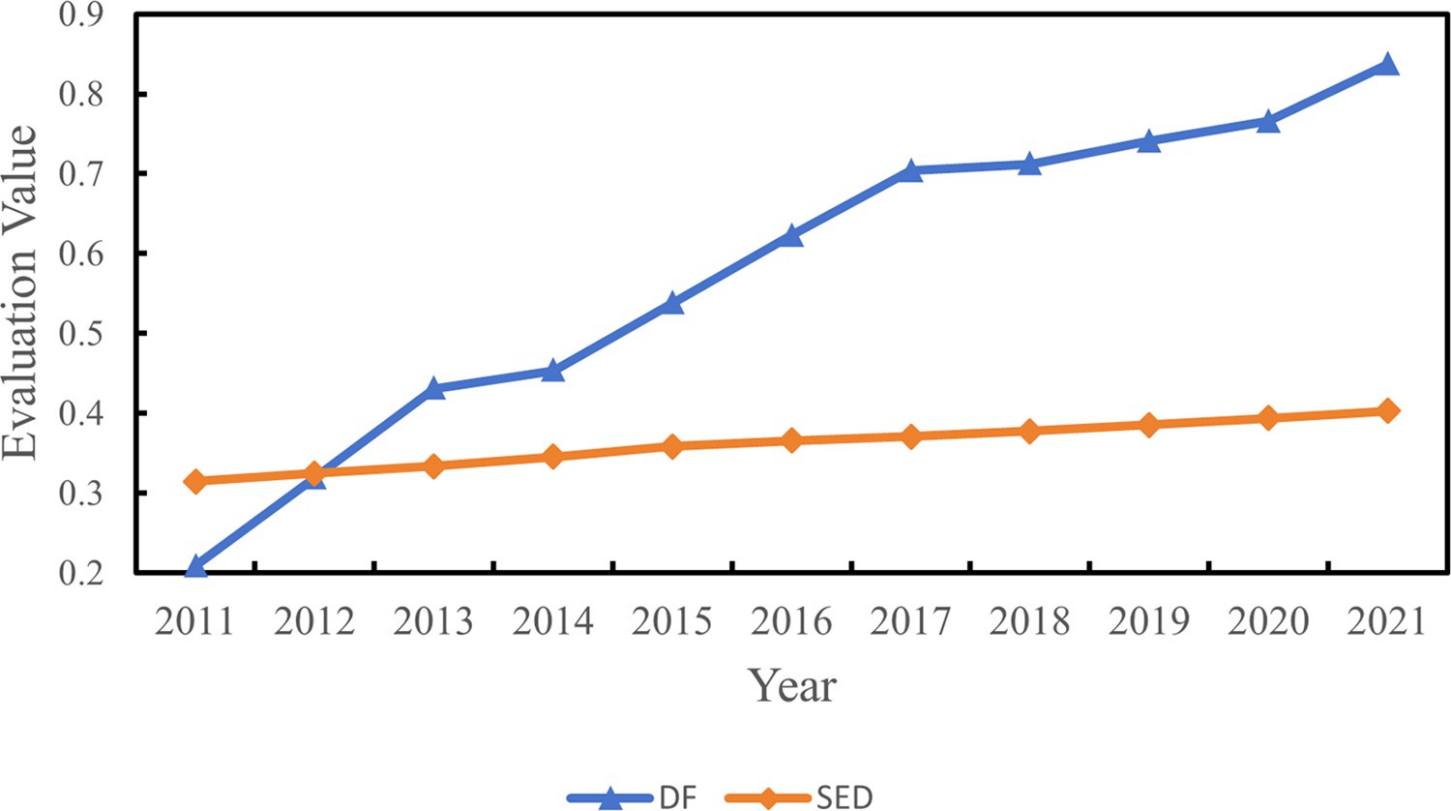
The evaluation value of DF and SED in the YRB.

The entropy method is applied here to evaluate the level of DF and SED of 55 cities in the YRB from 2011 to 2021. The corresponding outcomes are summarized in Fig 4.

Examining the progression of DF across various regions over the years, there has clearly been a notable surge in DF advancement during the analyzed timeframe. The sample regions showcased a substantial overall improvement of 300.88% over the course of eleven years. Among the sub-regions, the upstream areas experienced the most significant enhancement, with a remarkable increase of 302.38%. Meanwhile, the downstream areas witnessed a comparatively smaller enhancement of 299.60%. The average DF value across all sampled cities in the YRB stands at 0.5757, accompanied by a mean yearly growth rate of 13.45% throughout the examination period. The specific results of the upstream, midstream, and downstream areas are shown in Table 2.

**Table 2:** Level of DF and SED in the three regions (selected years).

systems	Regions	2011	2013	2015	2017	2019	2021	average
DF	Upstream	0.2005	0.4204	0.5315	0.6924	0.7096	0.8069	0.5596
	Midstream	0.2082	0.4224	0.5351	0.699	0.74	0.8345	0.5721
	Downstream	0.2178	0.4508	0.5476	0.7194	0.7729	0.8703	0.5956
SED	Upstream	0.2929	0.3159	0.3446	0.3618	0.3772	0.3928	0.3479
	Midstream	0.2882	0.3062	0.3309	0.342	0.3599	0.378	0.3347
	Downstream	0.3639	0.3796	0.401	0.4097	0.4201	0.4391	0.4022

From the perspective of SED, progress was lackluster throughout the examined period, characterized by low growth rates both in the full sample and within each region. The overall growth over the eleven years amounted to 28.09%, with a mean yearly growth rate of 2.28%. At the sub-regional level, as shown in Table 2, the upstream areas demonstrated the highest improvement, at 34.13%; the downstream areas exhibited the smallest improvement, at 20.66%. Throughout the eleven years, the mean value of the full sample was 0.3611, with the downstream areas having the largest mean value of 0.4022; the midstream areas showed the lowest mean value of 0.3347. Overall, the level of DF in the YRB surpassed the level of SED as a whole.

#### 3.1.2 System CCD results and analysis

Drawing upon the foundation of the CCD model, an evaluation of the interplay and coordination between DF and SED in the YRB is now conducted. This assessment encompasses both the full sample and individual regions within the YRB, spanning the period from 2011 to 2021. The comprehensive findings, which fully depict the outcomes, are presented in Table 3.

**Table 3:** The CCD in the YRB as a whole and in each region.

Year	Full sample		Upstream		Midstream		Downstream	
	CCD	Grade	CCD	Grade	CCD	Grade	CCD	Grade
2011	0.502	Near	0.4871	Near non	0.4915	Near non	0.5278	Near
2012	0.5633	Near	0.5525	Near	0.5489	Near	0.5891	Near
2013	0.6113	Primary	0.5986	Near	0.5961	Near	0.64	Primary
2014	0.6249	Primary	0.6177	Primary	0.6092	Primary	0.6486	Primary
2015	0.6588	Primary	0.6504	Primary	0.6451	Primary	0.6817	Primary
2016	0.687	Primary	0.6782	Primary	0.6732	Primary	0.7103	Intermediate
2017	0.7111	Intermediate	0.7036	Intermediate	0.6961	Primary	0.7344	Intermediate
2018	0.717	Intermediate	0.7043	Intermediate	0.7043	Intermediate	0.7432	Intermediate
2019	0.7279	Intermediate	0.7161	Intermediate	0.7154	Intermediate	0.7528	Intermediate
2020	0.7382	Intermediate	0.7251	Intermediate	0.7268	Intermediate	0.7634	Intermediate
2021	0.7594	Intermediate	0.7474	Intermediate	0.747	Intermediate	0.7844	Intermediate
Average	0.6637	Primary	0.6528	Primary	0.6503	Primary	0.6887	Primary

The CCD of the full sample and each region steadily increased during the sample period. The CCD of the full sample has also been adjusted from the near coordination level in 2011 to the primary coordination level in 2021. As of 2021, the YRB as a whole and the three regions were all at the primary coordination level. When considering sub-regions, the CCD of the downstream areas consistently surpassed the average level of the YRB as a whole in all the sampled years. In contrast, the CCD of the upstream areas closely aligned with the overall average level of the YRB and slightly lagged behind that level.

### 3.2 Evolution of the distributional dynamics of CCD

#### 3.2.1 Kernel density estimation

**Fig 5:**
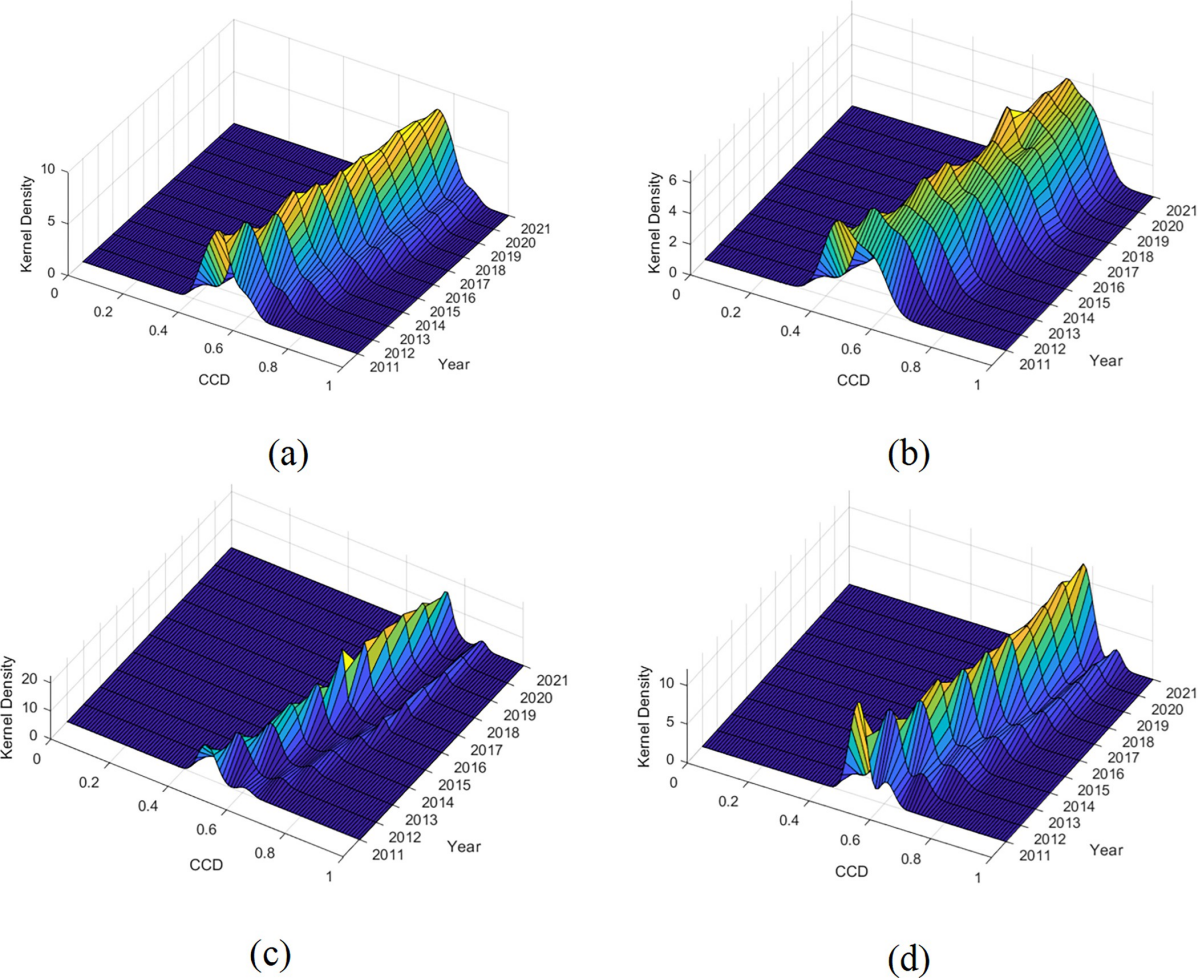
Kernel density estimation of the CCD.

Owing to disparities in factor endowments and developmental stages among the upstream, midstream, and downstream areas, the measurement outcomes and respective trends of each region may exhibit temporal variations. Consequently, considering the distribution location and pattern, this paper employs kernel density estimation [43] to scrutinize the dynamic evolution of the CCD for both the full sample and the three regions. The outcomes of this analysis are visually depicted in Fig 5.

Fig 5(A) shows the dynamic evolution trend of the CCD in the full sample. When considering the distribution location, the center of the overall distribution curve and the change interval gradually shifted to the right. However, the rate of movement gradually decelerated over the years and eventually reached a point of relative stagnation after 2017. When examining the distribution pattern, a general upward trend can be observed in the height of the primary peak of the distribution curve, while the development course generally shows the M-shaped evolution characteristics of “rising-falling-rising-stable”. Simultaneously, the width of the main peak contracted, indicating that the coupling coordination level of the full sample was centralized. This was accompanied by a reduction in regional disparities.

Figs 5(B)–5(D) depict the dynamic evolutionary trends of the CCD between DF and SED in the upstream, midstream, and downstream, respectively, of the YRB during the sample period. First, regarding distribution location, the evolutionary trend of the three sampled regions aligns with the overall distribution of the full sample, which was generally on the rise. However, the upstream and midstream areas fell back somewhat in the years 2018–2020. Second, from the distribution pattern, except for the upstream area, the midstream and downstream areas gradually evolved in the form of two obvious peaks, indicating that the regional differences in the change of CCD were getting bigger.

#### 3.2.2 Markov chain analysis

**Fig 6:**
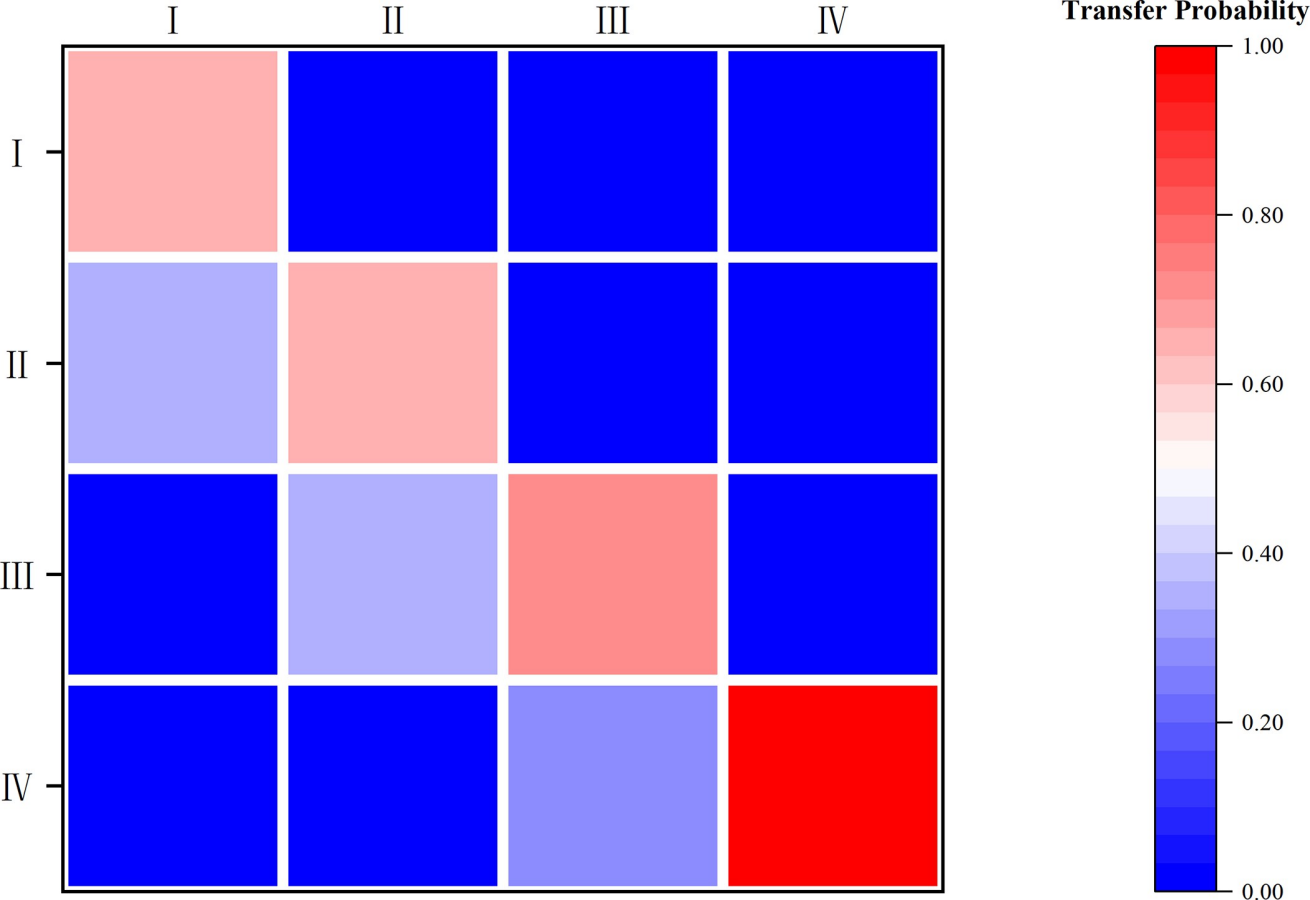
Traditional MTPM for the CCD.

In order to provide further insight into the internal dynamics and spatial transfer characteristics of CCD, this study introduces the MTPM for analysis. Initially, a conventional Markov chain is utilized to assess the intrinsic trend characteristics of the system’s CCD. The quartile method is employed to classify the CCD levels of the sampled cities into four distinct grades, based on their respective average magnitudes: low, medium-low, medium-high, and high. The resulting transfer probability matrix is obtained, as depicted in Fig 6. Notably, the diagonal elements consistently surpass the non-diagonal elements, in which the probability of maintaining the original level after one year for the areas in the low, medium-low, medium-high, and high levels is 65.56%, 64.47%, 71.11%, and 100.00%, respectively. This finding indicates that the CCD is relatively stable among different levels, the phenomenon of “club convergence” has appeared. Moreover, the overall probability of convergence of low and high levels is slightly higher than that of medium-low and medium-high levels, indicating that there is a certain degree of the "Matthew effect" within the system’s CCD.

#### 3.2.3 Spatial Markov chain analysis

To explore the spatial interdependence and diffusion of CCD in the YRB, using Stata 17.0 software, the spatial Moran’s I index of CCD is computed from 2011 to 2021. The measured CCD data and corresponding results are presented in Table 4. The computed outcomes consistently exhibit a significantly positive global Moran’s I index for CCD within the YRB throughout the examination period. This observation suggests that the CCD level is influenced by the CCD levels of neighboring regions. At the same time, a high-high and low-low aggregation is shown. The values of Moran’s I index range consistently between 0.1 and 0.3, indicating a relatively stable spatial positive correlation among CCD levels.

**Table 4:** Global Moran’s index of the CCD (selected years).

	2011	2013	2015	2017	2019	2021
Moran’s I	0.217***	0.239***	0.198***	0.205***	0.191**	0.215***
Z-value	2.567	2.815	2.371	2.444	2.296	2.553

Note: Owing to spatial limitations, the measurements presented herein are exclusively reported for specific years; with ***, **, and * indicating significance at the 1%, 5%, and 10% levels, respectively.

**Fig 7:**
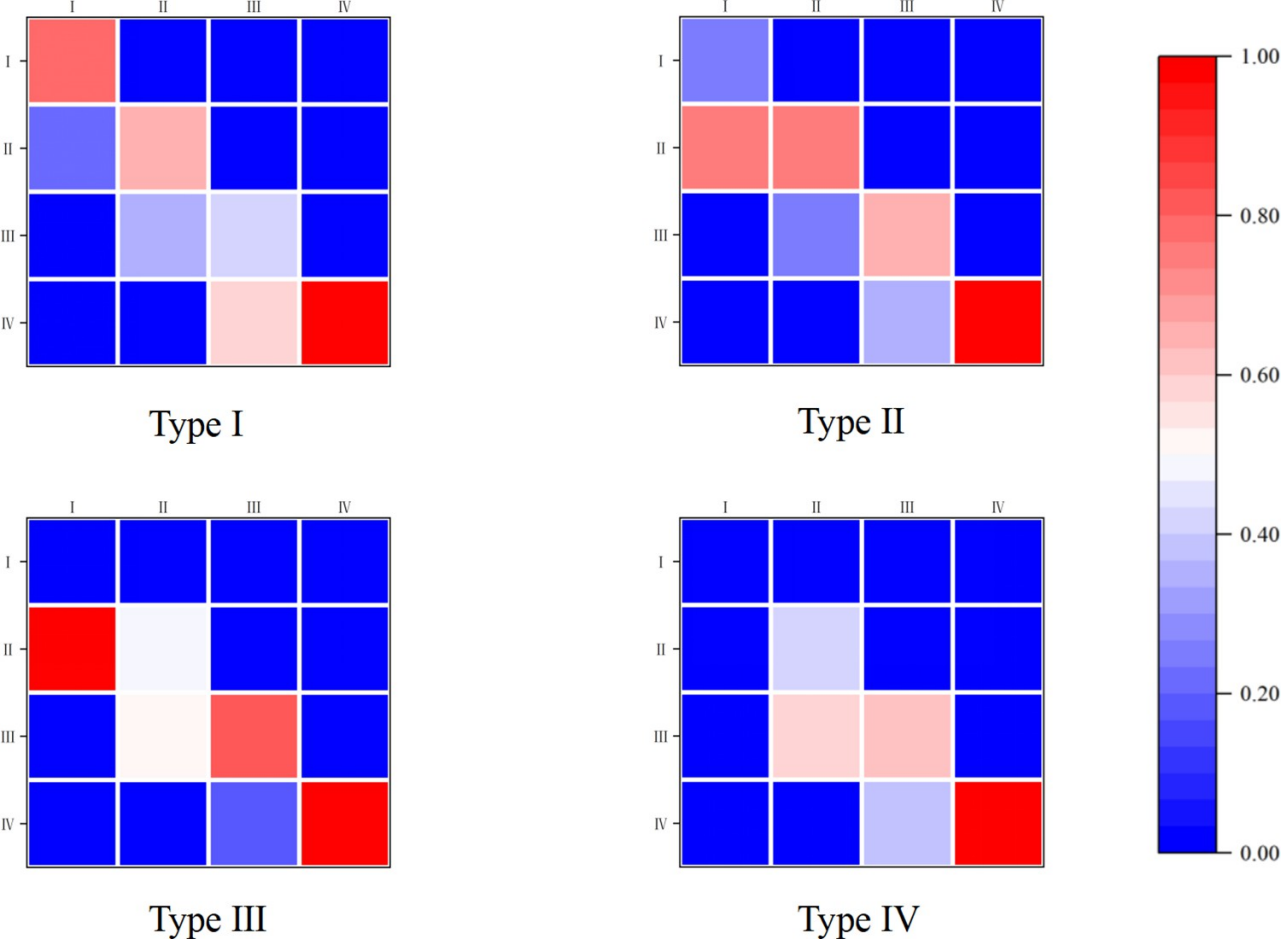
Spatial MTPM for the CCD of DF and SED.

The above results indicate that the spatial factor must be taken into account to establish the spatial MTPM; the results are shown in Fig 7. Firstly, the four transfer probability matrices exhibit variations across different spatial lag types. This implies that the probability of the transfer occurring due to the influence of coupling coordination in the region varies in the case of differences in coupling coordination in neighboring regions. Secondly, under different spatial lag types, the diagonal elements of the transfer probability matrices do not consistently surpass the non-diagonal elements. This suggests that the likelihood of coupling coordination being “rank-locked” decreases under the spatial spillover effect. In addition, non-zero elements are present on both sides of the diagonal. This implies that the CCD is unstable, although the CCD can realize an upward transfer under ideal conditions. There is also a certain risk of downward transfer, and there is only the rank transfer of adjacent types. It is also difficult to realize a cross-level jump. Further, different lag types have different effects on the same rank. The likelihood of transferring from low to medium-low levels under medium-high level lag types is 100%, which is clearly significantly greater than the probability of transferring under low level lag types. Lastly, the impact of the same lag type varies across levels. Under medium-high level lag conditions, the likelihood of an upward transfer of one level for low, medium-low, and medium-high levels is 100%, 51.02%, and 19.18%, respectively. Clearly, this shows a decreasing trend, suggesting that the transfer probability is influenced by both the lag type and the initial level of coupling coordination.

## 4 Convergence analysis

### 4.1 *σ*-convergence result analysis

**Fig 8:**
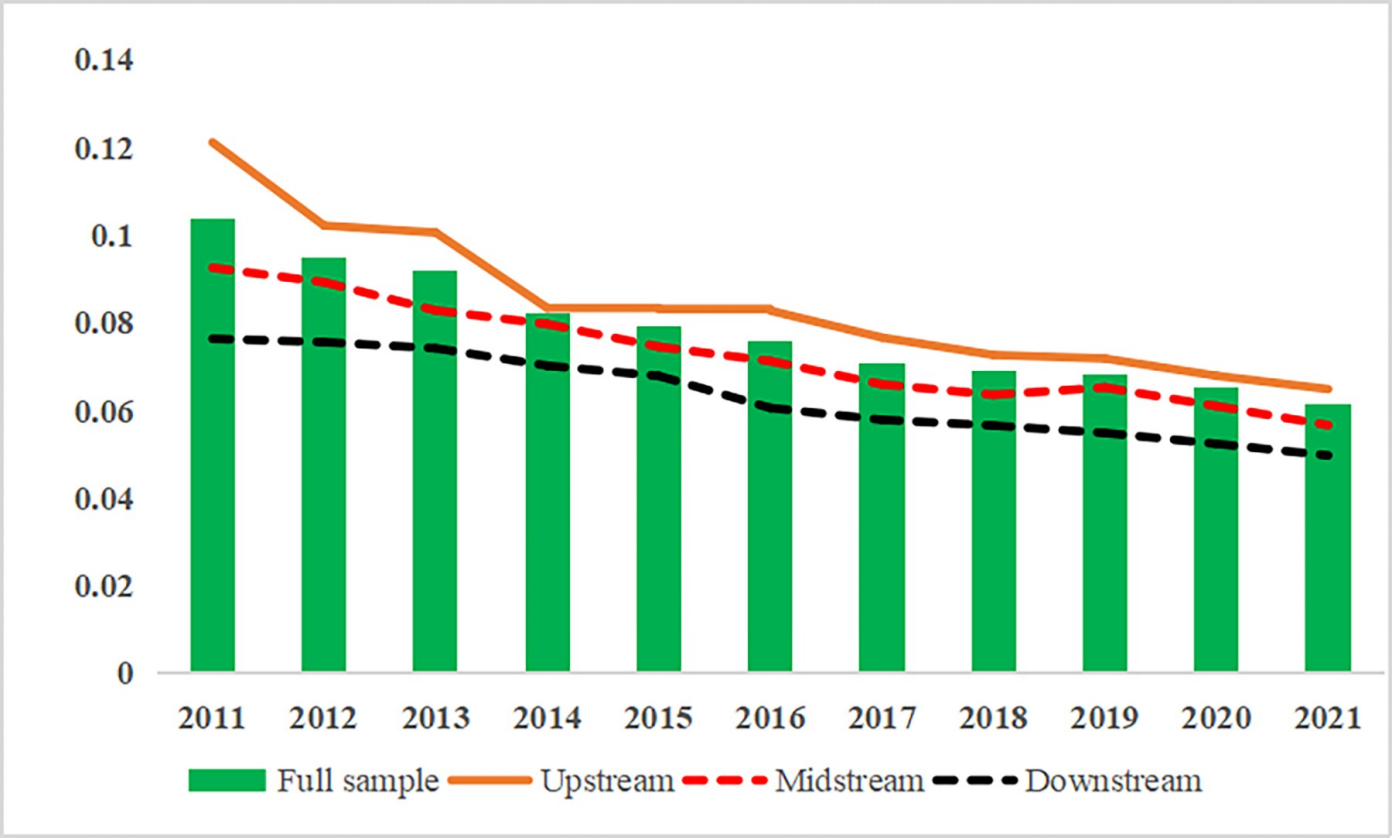
Graphical representation of *σ*-convergence of the CCD.

This paper adopts the coefficient of variation for a *σ*-convergence test and measures the coefficient of variation of CCD from 2011 to 2021. The convergence trend is plotted and, as shown in Fig 8, the coefficient of CCD exhibited a stable year-by-year decreasing trend. This finding indicates that the CCD of the YRB as a whole exhibited a typical *σ*-convergence. Sub-regionally, the most significant decline in the coefficient was observed in the upstream areas, surpassing the overall YRB sample level. The decrease of coefficient in the midstream areas was the second largest, and the convergence was a little weak; its trend was also closer to the full sample of the YRB. The decrease of the coefficient in the downstream areas was smaller and had the lowest rate of convergence.

### 4.2 *β*-convergence result analysis

#### 4.2.1 Absolute *β*-convergence analysis

As mentioned earlier, due to the substantial positive spatial correlation between the CCD of DF and SED, the spatial factor is included here to discuss the convergence characteristics of CCD. The LM test concludes that the spatial Durbin model (SDM) has better validity. In addition, the Hausman test rejects the original hypothesis of the random effect. This article finally selects the two-way fixed effect SDM model to test the *β*-convergence process of the CCD.

Table 5 reveals the test outcomes of absolute *β*-convergence for both the full sample and the three regions, and the estimated coefficient is always obviously significantly negative. This finding indicates an absolute *β*-convergence characteristic of the CCD for both the full sample and the three regions. From the convergence results, the convergence speed and halfway convergence period of each region can be seen to be different. The convergence speeds of the full sample and three regions are 6.39%, 9.47%, 4.37%, and 4.11%, respectively; the corresponding halfway convergence periods are 10.85 years, 7.32 years, 15.88 years, and 16.87 years, respectively. Among them, the convergence rate of the upstream areas is higher than that of the YRB as a whole, and the corresponding halfway convergence period is the shortest, at 7.32 years. Furthermore, in order to fit the actual development situation, the conditional *β*-convergence of the CCD for both the full sample and the three regions is examined under the consideration of the control variables.

**Table 5:** Absolute *β*-convergence of the CCD.

Variables	Full sample	Upstream	Midstream	Downstream
*β*	-0.4723*** (-17.60)	-0.6123*** (-12.38)	-0.3538*** (-9.56)	-0.3369*** (-6.38)
City fixed	Yes	Yes	Yes	Yes
Time fixed	Yes	Yes	Yes	Yes
R^2^	0.5206	0.5331	0.6749	0.3220
Convergence rate (%)	6.39	9.47	4.37	4.11
Halfway convergence period (year)	10.85	7.32	15.88	16.87
N	550	180	190	180

Note: *, **, and *** denote the significance levels of 10%, 5%, and 1%, respectively, with t-statistics in parentheses.

#### 4.2.2 Conditional *β*-convergence analysis

In order to examine the potential factors that influence the CCD, referring to the research conducted by Yao et al. (2022) [44], this study incorporates five control variables, as follows: GDP per capita (X1): The GDP per capita is employed as a measure of each city’s economic development, and the data are adjusted for inflation using 2011 as the base year. Urbanization rate (X2): The urbanization rate of each city reflects its degree of urbanization. Advancement of industrial structure (X3): The proportion of the tertiary industry output value to the secondary industry output value is employed as a proxy indicator. Number of software employees (X4): This variable is measured by the ratio of employees engaged in information transmission, computer services, and software to the resident population. Market openness (X5): The number of foreign-invested industrial enterprises above the scale is selected as a proxy variable for the openness of the city.

The estimation results of spatial conditional *β*-convergence after adding the above control variables are shown in Table 6. The coefficients for both the full sample and the three regions are significantly negative, indicating that CCD shows significant *β*-convergence. Specifically, the convergence speeds were 8.35%, 12.00%, 4.44%, and 17.69%, and the corresponding halfway convergence periods were 8.30 years, 5.78 years, 15.60 years, and 3.92 years, respectively. It is evident that, after considering the influence of factors such as GDP per capita, the convergence speed of the YRB as a whole and the three individual regions increased to different degrees. Also, the halfway convergence period shortened. The aforementioned outcomes indicate that the introduction of control variables promotes the *β*-convergence speed of CCD. In addition, the order of the convergence speed of the full sample and the three regions also changed, showing the characteristics of downstream>upstream>full sample>midstream.

**Table 6:** Conditional *β*-convergence of the CCD.

Variables	Full sample	Upstream	Midstream	Downstream
*β*	-0.5662*** (-20.01)	-0.6987*** (-13.64)	-0.3588*** (-8.88)	-0.8294*** (-13.74)
X1	3.25e-07*** (2.61)	1.31e-07 (0.58)	6.48e-07*** (3.06)	1.25e-07 (0.69)
X2	0.0336 (1.62)	0.0699** (2.05)	0.0604 (1.33)	0.0259 (0.49)
X3	0.0095*** (4.18)	0.0131*** (3.60)	0.0073 (1.52)	0.0073 (1.40)
X4	0.0074** (2.00)	0.0002 (0.03)	0.0081 (1.15)	-0.0019 (-0.43)
X5	0.0006*** (5.83)	0.00003 (0.05)	0.0004 (1.10)	0.0010*** (10.92)
City fixed	Yes	Yes	Yes	Yes
Time fixed	Yes	Yes	Yes	Yes
R^2^	0.7000	0.7018	0.7360	0.6928
Rate of convergence (%)	8.35	12.00	4.44	17.69
Halfway convergence period (year)	8.30	5.78	15.60	3.92
N	550	180	190	180

Note: *, **, and *** denote significance levels of 10%, 5%, and 1%, respectively, with t-statistics in parentheses.

## 5 QAP analysis

### 5.1 QAP correlation analysis

To get the results of the QAP correlation analysis between regional differences in CCD and the explanatory variables, this study used Ucinet software to carry out 2000 times of random replacement. The results are shown in Table 7. The outcomes reveal that the correlation coefficients between the regional differences in CCD and the regional differences in GDP per capita, urbanization rate, advancement of industrial structure, number of software employees, and market openness are all positive. In addition, the correlation coefficients of the other variables (except for the advancement of industrial structure) are all significant at the 1% level. This finding suggests that a difference in the development of any one of the factors will cause changes in the difference in CCD. The correlation coefficients of regional differences in GDP per capita, urbanization rate, number of software employees, and market openness with regional differences in CCD are 0.650, 0.797, 0.760, and 0.714, respectively.

**Table 7:** QAP correlation analysis results.

Variable	CCD	X1	X2	X3	X4	X5
CCD	1.000***	-	-	-	-	-
X1	0.650***	1.000***	-	-	-	-
X2	0.797***	0.749***	1.000***	-	-	-
X3	0.182*	-0.230**	0.017	1.000***	-	-
X4	0.760***	0.448***	0.631***	0.319**	1.000***	-
X5	0.714***	0.317**	0.321*	0.150	0.610***	1.000***

Note: *, **, and *** denote significance levels of 10%, 5%, and 1%, respectively.

### 5.2 QAP regression analysis

To analyze the extent and direction of the effect of the five abovementioned factors, 2000 permutations were selected for random replacement; QAP regression was conducted with the matrix of regional differences in CCD as the dependent variable and the matrix of regional differences in the five variables as the explanatory variable. The outcomes are presented in Table 8. In the regression results, the adjusted coefficient of determination is 0.883, indicating that these five difference matrices can explain 88.3% of the CCD regional differences. Based on the findings presented in the table, the standardized regression coefficients obviously eliminate the influence of the magnitude, compared with the non-standardized regression coefficients. In addition, the standardized regression coefficients of different variables can be directly compared [45]. Therefore, the standardized regression coefficients were applied to assess the effect of each factor on the regional differences in CCD. The consequential findings are as follows: (1) The coefficient of regional differences in the number of software employees is 0.071, but that figure is not significant, indicating that the regional differences in the number of software employees are not currently the main factor causing regional differences in CCD. (2) The standardized regression coefficients of regional differences in GDP per capita, urbanization rate, advancement of industrial structure, and market openness are all significantly positive. This finding indicates that these variables are the main factors causing regional differences in CCD. Among them, the regional difference in urbanization rate has the greatest effect on the regional difference in CCD, with a regression coefficient of 0.525. This indicates that an expansion of the regional difference in the level of urbanization will lead to an expansion of the regional difference in CCD. The intensity of the influence of other variables is in the order of: market openness, GDP per capita, and advancement of industrial structure. Of those variables, the standardized regression coefficient of market openness is 0.452; the standardized regression coefficients of GDP per capita and advancement of industrial structure are both 0.107.

**Table 8:** QAP regression results of factors affecting regional differences in CCD.

Variable	Unstandardized regression coefficients	Standardized regression coefficient	Probability of significance	Probability A	Probability B
Intercept	0.000	0.000			
X1	0.000	0.107	0.088	0.088	0.912
X2	0.198	0.525	0.000	0.000	1.000
X3	0.012	0.107	0.043	0.043	0.957
X4	0.020	0.071	0.175	0.175	0.826
X5	0.001	0.452	0.000	0.000	1.000

## 6 Discussion

This study measures the DF and SED level of 55 cities in the YRB, while also measuring their CCDs. Under the background of the global promotion of green, sustainable development, the analytical conclusions of this paper help to grasp the coupling coordination level and dynamic evolution trend of DF and SED in the YRB. This research also provides a theoretical basis and policy suggestions for promoting the coupling coordination level of DF and SED, both now and in the future. The findings of this paper therefore deserve further in-depth discussion.

Firstly, both the level of DF and SED witnessed continuous improvement during the sampled years. During that observed period, DF and SED grew by 300.96% and 28.11%, respectively. This growth can be attributed to the development of information technologies, such as big data and artificial intelligence, as well as government policy support. In recent years, the Chinese government has vigorously developed the digital economy [46], promoting its deep integration with the real economy. In particular, DF can better support industrial transformation and upgrading. However, SED is a comprehensive concept. The indicator system constructed in this study consists of various aspects and encompasses five dimensions: economic vitality, innovation power, green development, open development, and shared development. Although the government is actively promoting SED, many cities in the YRB are inland areas with relatively weak economic foundations and lower levels of openness. The YRB also faces additional challenges, such as a fragile ecological environment and severe pollution emissions [47]. Therefore, in the YRB, the level of SED is significantly lower than the level of DF.

Secondly, from a temporal perspective, CCD showed a stable growth trend from 2011 to 2021. Specifically, from 2011 to 2017, CCD experienced rapid growth mainly due to the fact that the early stage of the development of DF has a strong supportive effect on SED. For example, DF can alleviate financing constraints [48], facilitate the green transformation of industrial structures [49], and reduce industrial emissions of pollutants and carbon dioxide [50, 51], thereby effectively promoting SED. After 2018, CCD continued to grow but at a relatively slower pace. One possible reason for this could be that, after several years of rapid growth, DF and SED reached a higher level of coordination. The marginal contribution of DF to SED has slowed down, leading in turn to a slowdown in the growth rate of CCD. From the perspective of spatial evolution, most cities in the sample period underwent a transition from near and primary coordination to intermediate coordination. This indicates a significant improvement in the coordination between DF and SED in the YRB. Among them, the initial level in the upstream and midstream is near non-coordination, while the initial level in the downstream is near coordination. This is not difficult to understand, as the downstream area mainly includes cities in eastern China, such as Shandong Province and Henan Province. Compared to the upstream and middle areas, the downstream area is closer to the eastern coastal areas, has more abundant resources (such as labor, technology, and capital), and possesses a higher level of SED. All of this makes it easier for the downstream area to achieve coordinated development with DF.

Thirdly, the distribution of the CCD exhibits a non-random pattern and demonstrates spatial autocorrelation characteristics. From a spatial perspective, the spatial correlation test conducted in this study shows that CCD maintains stable positive spatial autocorrelation and exhibits significant high-high and low-low agglomeration. Previous studies have confirmed that a positive spatial correlation exits between the CCD of the digital economy and low-carbon development [52]. Theoretically, the development of the digital economy promotes the development of DF; low-carbon development can also be understood to be part of SED, which is similar to the focus of this study. The Chinese government has been advocating for regional coordinated development, and with the continuous improvement of the Internet and new digital infrastructure, the spatial and temporal distances between cities have become closer. For that reason, neighboring regions are more inclined to cooperate and exchange in areas such as economic construction, transportation infrastructure, ecological environment protection, and financial development [53]. This conclusion aligns with the research findings of this paper, which highlights the spatial patterns and relationships between DF and SED in the YRB.

Fourthly, this study examines the factors that influence regional differences in CCD. The results of the QAP regression indicate that regional differences in GDP per capita, urbanization rate, advancement of industrial structure, and market openness are the main factors contributing to CCD regional disparities. However, the regional differences in the number of software employees have no significant effect on CCD regional disparities. One possible reason for this is that, on the one hand, regional disparities in GDP per capita, urbanization rate, advancement of industrial structure, and market openness significantly influence the level of SED. On the other hand, although the number of software employees can affect the development of DF, their work is mainly concentrated in first-tier cities and other large cities. Cities in the YRB are mostly third-tier and fourth-tier cities with smaller economic scale and technological power, so they have relatively fewer software employees. Therefore, the differences between regions in the number of software employees do not have a significant impact on CCD disparities. Regarding the distribution of technology talents in China, the "Report on the Development of Science and Technology Talent in China 2022", compiled by the Ministry of Science and Technology, shows that technology talents are increasingly concentrated in eastern regions and a few central and western core cities. Thess cities are primarily concentrated in the Yangtze River Delta, the Pearl River Delta, and the Bohai Rim Economic Circle. Meanwhile, the talent outflow from underdeveloped regions in the northeastern and western areas has intensified.

In addition to answering the research questions mentioned above, this paper also provides a number of new contributions to related research. First, the research perspective of this paper is innovative, in that it offers a new quantitative analysis perspective. This study is the first to use a CCD model to analyze the dynamics between DF and SED in the YRB. This analysis helps relevant departments gain a better understanding of the coordinated development relationship between DF and SED. Second, this study further analyzes the expected changes and influencing factors of the spatial patterns of CCD between DF and SED. This analysis provides a basis upon which the Chinese government can formulate policies aimed at enhancing the coordinated development of DF and SED.

In addition to the aforementioned contributions, this study also has some limitations. Firstly, while the most recent data available are utilized, there is still a certain degree of lag as data updates occur. Further research should be conducted using updated data to enhance the accuracy and relevance of this study’s findings. Secondly, this study focuses solely on the YRB region as the research object. Considering the vast geographical scope of China, there are other city clusters, both large and small, that undoubtedly play significant roles in regional economic development. City clusters, such as those in the Yangtze River Economic Belt and the Pearl River Delta, should be considered as important areas for future research. Thirdly, as the basic administrative units in China’s local governance system, studying the level of DF and SED in county-level areas is of great practical significance and application value. However, a gap still exists in research related to county-level analysis, which is another limitation of this study. Exploring county-level dynamics should be pursued as an important direction and approach for future research.

## 7 Conclusions and policy implications

### 7.1 Conclusions

Based on the synergies between DF and SED, this study measures the DF and SED level of 55 cities in the YRB and then measures their CCDs. In addition, kernel density estimation, Markov chain, *σ*-convergence, *β*-convergence, and QAP methods are used to study the spatial pattern, distribution dynamic evolution trend, convergence, and influencing factors of the regional differences in the CCD. The main conclusions are as follows:

Firstly, during the sample period, the level of DF increased significantly, with an increase of 300.96%. Meanwhile, the level of SED grew more slowly, at a rate of only 28.11%. The CCD of the full sample and each region increased steadily during the sample period, and the full sample adjusted from the near coordination level in 2011 to the primary coordination level in 2021.

Secondly, the dynamic distribution pattern shows that the height of the main peak is generally on an upward trend, while the development process generally shows the M-shaped evolution characteristics of “rising-falling-rising-stable”. Simultaneously, the width of the main peak contracted, indicating that the CCD is centralized, and the differences between regions are shrinking. From the perspective of the spatial pattern, the spatial correlation test found the CCD had been having a stable positive spatial correlation during the sample period. Also, the CCD shows a significant high-high and low-low aggregation type.

Thirdly, in terms of convergence characteristics, the CCD between DF and SED shows both *σ*-convergence and spatial *β*-convergence. The analysis of spatial *β*-convergence shows the existence of both absolute *β*-convergence and conditional *β*-convergence. After introducing control variables (e.g., GDP per capita), the speed of convergence improved to different degrees, the halfway convergence period shortened, and the order of the speed of convergence presented the characteristics of downstream>upstream>full sample>midstream.

Fourthly, the QAP regression indicates the main reasons for the regional differences in CCD come from regional differences in GDP per capita, urbanization rate, advancement of industrial structure, and market openness. Meanwhile, the regional differences in the number of software employees have no significant effect on the regional differences in the CCD.

### 7.2 Policy implications

Drawing from the discussions and conclusions outlined above, it is evident that the CCD between DF and SED in the YRB is generally at a moderate level. However, certain underlying challenges persist that necessitate attention. In order to further promote the coordinated development of these two systems, this study proposes the following policy recommendations:

Firstly, there is a need to clarify the effective ways to improve SED in the YRB. Currently, the level of SED lags behind the development of DF. Based on the principles of harmonious coexistence between humans and nature, efforts should be made to promote sustainable development in the YRB, specifically in terms of greenness, innovation, and efficiency. The greatest weakness in the YRB lies in its ecological fragility. Therefore, there is a need to establish an ecological civilization value system and adhere to a green development model with low resource consumption. Secondly, technology innovation should be a focal point used to improve resource utilization efficiency in the YRB. Utilizing information technologies such as big data and artificial intelligence, emphasis should be placed on the development of high-tech and energy-saving industries. Thirdly, there is a need to expedite the transformation and upgrading of traditional industries. Due to factors such as natural resources and geographical location, the YRB is predominantly reliant on agriculture and manufacturing. Exploring its own characteristics and advantages is important to the YRB. For example, efforts should be made to develop rural tourism and modern green agriculture, continuously improving industrial efficiency.

Next, the coordination and interaction between DF and SED must be strengthened, in order to further promote the coordinated development of these two systems. Currently, the CCD between DF and SED in the YRB is at the intermediate level; there is still room for further improvement. Financial institutions should accelerate their digital transformation and play a greater role in supporting the green and low-carbon transformation of the YRB and the transformation and upgrading of traditional agriculture. They should also help meet the financing needs of small and micro enterprises. Additionally, financial institutions should utilize technologies such as big data and the Internet to effectively monitor and track the transformation and development of key industries with high emissions and pollution. Risks should be effectively managed, and the occurrence of systematic credit risk events should be reduced, thereby contributing to the SED of the YRB. On the other hand, the SED of the YRB will facilitate the construction of new infrastructure, promote progress in information technology, and drive the digitization of traditional financial industries. This, in turn, will contribute to the development of DF in the YRB.

Finally, the positive influences that affect the CCD of the two systems should be improved, and the coordinated development of the region should be steadily promoted. Regional disparities in per capita GDP, urbanization rate, industrial structure, and market openness can lead to differences in the CCD. First, human capital is the fundamental driving force for economic development. Cities in the YRB should continuously optimize population policies to attract high-tech talents and professionals, while simultaneously reducing the outflow of local talents. Additionally, efforts should be made to improve the level of education, achieving dual enhancement in both population quantity and quality. Second, the government should strengthen internal and external exchanges and cooperation. Internally, cities in the YRB should enhance information connectivity and infrastructure interconnection, thereby improving the driving capacity of provincial capital cities such as Xi’an, Taiyuan, Zhengzhou, and Jinan to surrounding cities. Simultaneously, continuous efforts should be made to strengthen external exchanges and openness, attracting advanced technology and capital. Through cooperative exchanges, regional disparities can be continuously narrowed, and SED can be achieved.

## Supporting information

S1 Dataset (XLSX)
